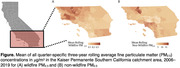# Long‐Term Wildfire Smoke Exposure and Incident Dementia in a Large California Cohort

**DOI:** 10.1002/alz.086179

**Published:** 2025-01-09

**Authors:** Holly C Elser, Timothy B Frankland, Sara Y Tartof, Elizabeth Rose Mayeda, Jennifer J. Manly, Jacqueline M Torres, Alexander J Northrop, Tarik Benmarhnia, Chen Chen, Joan A Casey

**Affiliations:** ^1^ Hospital of the University of Pennsylvania, Philadelphia, PA USA; ^2^ Kaiser Permanente Hawaii Center for Integrated Healthcare Research, Honolulu, HI USA; ^3^ Kaiser Permanente Southern California, Pasadena, CA USA; ^4^ University of California, Los Angeles Fielding School of Public Health, Los Angeles, CA USA; ^5^ Department of Neurology, Columbia University, New York, NY USA; ^6^ University of California San Francisco, San Francisco, CA USA; ^7^ Vagelos College of Physicians and Surgeons at Columbia University, New York, NY USA; ^8^ Scripps Institution of Oceanography, University of California, San Diego, CA USA; ^9^ Scripps Institution of Oceanography, UC San Diego, La Jolla, CA USA; ^10^ Univeristy of Washington School of Public Health, Seattle, WA USA

## Abstract

**Background:**

Long‐term exposure to ambient air pollution–including fine particulate matter <2.5µm in diameter (PM_2.5_)–has previously been associated with incident dementia. As climate change drives longer and more intense wildfire seasons, exposure to PM_2.5_ produced by wildfires may be a unique and increasingly important risk factor for dementia.

**Method:**

In this retrospective open cohort study, we examined the association between long‐term exposure to wildfire PM_2.5_ and dementia among Kaiser Permanente Southern California patients aged ≥60 from 2009‐2019 in California. Study participants were dementia‐free at baseline. Incident dementia was identified within the electronic health record using *International Classification of Diseases* 9 and 10 codes. Estimates for rolling three‐year average concentrations of wildfire and non‐wildfire PM_2.5_ were assigned for each participant based on census tract of residence, which was updated quarterly. We used pooled logistic regression to estimate the odds of dementia diagnosis associated with a one µg/m^3^ increase in the three‐year average of wildfire and non‐wildfire PM_2.5_. All models included fixed effects for calendar year and adjusted for age, sex, race and ethnicity, marital status, smoking status, Charlson Comorbidity Index (minus dementia), and census tract poverty and population density.

**Result:**

The study included 1,227,241 members. Approximately half were women (53%) and were married (54%). A majority self‐identified as non‐Hispanic White (49%) or Hispanic (26%). Over the study period, the mean wildfire PM_2.5_ concentration was 0.09 (IQR: 9.6‐12.4). After adjusting for covariates, the odds of dementia diagnosis was 10% higher for every 1 µg/m^3^ higher three‐year average wildfire PM_2.5_ concentration (OR = 1.10, 95%CI: 0.96,1.25). For non‐wildfire PM_2.5_ the odds of dementia diagnosis was 1% higher for every 1 µg/m^3^ higher three‐year average exposure (OR = 1.01, 95%CI: 1.00,1.01).

**Conclusion:**

Long‐term exposure to PM_2.5_, both wildfire and non‐wildfire, may be an important risk factor for dementia.